# Depressive and anxiety symptoms among college students in Anhui Province, China: three repeated cross-sectional surveys

**DOI:** 10.3389/fpubh.2026.1917300

**Published:** 2026-09-15

**Authors:** Xiaomei Gao, Siqi Mu, Wanrong Wang, Yan Ru, Chengcheng Zhang, Ping Li, Juan Zhang, Yichang Du, El Jaidi Imane, Qiu Zhang, Honglin Hu, Fang Dai, Peng Wang, Yi Zhang

**Affiliations:** 1 The First Affiliated Hospital of Anhui Medical University, Hefei, China; 2 Department of Endocrinology, Sir Run Run Shaw Hospital, Zhejiang University School of Medicine, Hangzhou, Zhejiang, China; 3 Inflammation and Immune Mediated Diseases Laboratory of Anhui Province, Key Laboratory of Anti-inflammatory of Immune Medicines, Ministry of Education, Anhui Institute of Innovative Drugs, School of Pharmaceutical Sciences, Anhui Medical University, Hefei, China; 4 School of Life Sciences, Anhui Medical University, Hefei, China; 5 School of Nursing, Anhui Medical University, Hefei, Anhui, China

**Keywords:** anxiety, college students, depression, GAD-7, mental health, PHQ-9, questionnaire survey

## Abstract

**Background:**

Depression and anxiety symptoms are prevalent among college students and significantly affect their academic performance, interpersonal functioning, and long-term well-being. Repeated cross-sectional surveys can reveal temporal variations in symptom burden, yet rigorous data harmonization is essential to ensure valid cross-year comparisons. This study aimed to harmonize three waves of questionnaire survey data and provide a comprehensive descriptive summary of depressive and anxiety symptoms among Chinese college students.

**Methods:**

Questionnaire submissions were recorded from 9 November 2023 to 3 January 2024, 14 May to 14 June 2024, and 7 April to 5 May 2025. Student identifiers were used to identify repeated within-wave submissions and overlap across administrations. After retaining the earliest response per student within each administration, 4,236 records remained (2023: 1,998; 2024: 1,743; 2025: 495). Depressive and anxiety symptoms were measured using the PHQ-9 and GAD-7. Inter-administration comparisons used Kruskal-Wallis and chi-square tests with effect sizes. Generalized estimating equations accounted for repeated observations of the same student. Internal consistency, PHQ-9/GAD-7 convergence, and multivariable correlates of PHQ-9 Item 9 endorsement were evaluated.

**Results:**

The analytic dataset included 4,236 wave-specific records from 2,782 unique students; 240 students participated in all three administrations. Overall mean PHQ-9 and GAD-7 scores were 5.08 ± 4.08 and 3.21 ± 3.70, respectively. Differences across administrations were statistically significant (PHQ-9 epsilon-squared = 0.006; GAD-7 epsilon-squared = 0.007). In age- and sex-adjusted GEE models, the 2024 administration had higher PHQ-9 scores than 2023 (adjusted mean difference 0.49, 95% CI 0.24–0.74), whereas the 2025 difference was not significant. PHQ-9 and GAD-7 demonstrated high internal consistency (alpha = 0.890 and 0.937) and strong convergence (Spearman rho = 0.798).

**Conclusion:**

Mild depressive and anxiety symptoms were highly prevalent in this college cohort, while moderate-to-severe symptoms affected a clinically significant minority. The temporal peak in 2024 and the persistent elevation of self-harm ideation in 2025, underscore the necessity of a tiered campus mental health strategy and the critical importance of independent, continuous suicide risk surveillance. The harmonized dataset provides a robust foundation for future longitudinal risk-factor modeling and evidence-based intervention planning.

## 1 Introduction

The mental health issues of college students are an urgent public health and education problem worldwide (1, 2). During the transition to university life, factors such as increased academic demands, the development of social networks, changes in living environments, enhanced personal autonomy, and uncertainty in future career paths may create a period where college students are more susceptible to emotional distress (3). At this stage of development, students may be particularly susceptible to symptoms of depression and anxiety, especially when they experience insufficient social support, poor sleep habits, low levels of physical activity, addiction to virtual networks, smoking or drinking. Therefore, understanding the burden and temporal changes of such symptoms in continuous student populations is a key first step toward achieving effective campus mental health prevention and early intervention (4, 5).

Depressive and anxiety symptoms warrant particular attention because they can adversely affect academic performance, interpersonal functioning, sleep quality, physical activity engagement, and long-term health trajectories (6). Even mild symptom levels can be clinically meaningful in student populations due to their high prevalence and their potential to identify individuals who may benefit from monitoring, psychoeducation, or low-intensity supportive interventions (7, 8). Consequently, systematic, repeated cross-sectional characterization of symptom burden across multiple survey waves can provide actionable insights for university mental health services and inform the design of tiered prevention strategies (9).

The present study used three serial questionnaire administrations covering sociodemographic characteristics, family context, health behavior, physical activity, digital media use, sleep, the nine-item Patient Health Questionnaire (PHQ-9), and the seven-item Generalized Anxiety Disorder scale (GAD-7) (10, 11). In response to concerns regarding design classification and data quality, student identifiers were used to detect within-wave duplicate submissions and quantify overlap between administrations. The corrected analysis incorporated inferential testing, effect sizes, cluster-aware models, psychometric evaluation, and focused analysis of PHQ-9 Item 9 (12, 13). The present manuscript focuses on: (1) describe depression and anxiety symptom burden across three survey administrations; (2) determine whether observed differences exceeded sampling variability and quantify their magnitude; (3) evaluate PHQ-9 and GAD-7 internal consistency and convergent association; and (4) characterize students endorsing self-harm-related thoughts and identify associated factors. The findings are intended to support transparent campus mental health surveillance and may offer a transferable framework for universities in other settings.

## 2 Methods

### 2.1 Study design and participants

This study comprised three serial questionnaire administrations. The first recorded submissions occurred on 9 November 2023, 14 May 2024, and 7 April 2025, and the collection windows ending on 3 January 2024, 14 June 2024, and 5 May 2025, respectively. The source files contained 2,129, 1,770, and 552 submitted records. Student identifiers were normalized as character strings and matched across files. Within each administration, repeated submissions under the same identifier were identified. To avoid multiple counting of the same student within a survey administration, the earliest completed response was retained. This procedure removed 215 repeated submissions and yielded 4,236 wave-specific analytic records: 1,998 in 2023, 1,743 in 2024, and 495 in 2025. Across administrations, 1,002 students were observed in both 2023 and 2024, 393 in both 2023 and 2025, 296 in both 2024 and 2025, and 240 in all three administrations. In total, the analytic dataset represented 2,782 unique students. The 2025 source file was labeled as a follow-up of the 2023-entry cohort, and student-ID matching confirmed substantial overlap across administrations. Therefore, the sample for 2025 becomes a relatively narrower follow-up sample. Student identifiers predominantly corresponded to the 2023-entry cohort, but grade distribution was not reconstructed beyond the information supported by the source files.

The present manuscript was developed using a processed and harmonized analytical dataset rather than the original questionnaire export files. During data preprocessing, variable names, response options, and coding schemes were systematically standardized across survey waves to ensure consistency and comparability. All variable labels and categorical response labels were translated into English to facilitate international dissemination and subsequent statistical analyses. In addition, to protect the privacy of participants, we removed direct personal identification information from the study and only retained an anonymized participant identifier for analysis of traceability and possible future association analysis. Because the primary objective of the current study was to describe the structure and characteristics of the processed dataset and to summarize mental health outcomes across survey waves, all analyses were descriptive in nature. Therefore, the findings should be interpreted as population-level descriptive observations rather than evidence of causal relationships between variables.

### 2.2 Questionnaire domains

This questionnaire aims to comprehensively assess various factors that may be related to the physical and mental health of college students. The survey covers demographic, family, behavioral, lifestyle, and psychological aspects, enabling a multi-dimensional description of students’ health status and laying the foundation for future epidemiological and risk factor analyses.

Demographic variables include gender, age or date of birth, grade level, height, weight, family residence, whether an only child, parental education level, family economic status, and number of close friends. These variables were collected to describe the sociodemographic composition of the study population and to provide contextual information for subsequent subgroup analyses. Health-related behavioral indicators include smoking, alcohol consumption, vigorous exercise, moderate-intensity exercise, walking behavior, sports participation, sedentary time, and non-academic digital media use. These behavioral factors are widely recognized as important determinants of the physical and mental health of young people and college students. Furthermore, this study also collected information on sleep habits and sleep-related problems. Sleep is closely related to mood regulation, cognitive ability, and mental health, and is therefore an important component of student health assessment.

Mental health status was assessed using standardized self-report scales that measure symptoms of depression and anxiety. Specifically, depressive symptoms were assessed using the PHQ-9, and anxiety symptoms were assessed using the GAD-7. These questionnaire domains collectively construct a comprehensive dataset that can be used for descriptive epidemiological studies, subgroup comparisons, mental health surveillance, and future multivariate analyses to explore the potential determinants of mental health in college students.

### 2.3 Assessment of depressive symptoms

Depressive symptoms were assessed using the PHQ-9, a standardized instrument commonly used for screening and quantifying depressive symptom burden (14). The PHQ-9 evaluates nine depressive symptom domains experienced during the previous 2 weeks, including loss of interest or pleasure, depressed mood, sleep disturbance, fatigue, appetite change, feelings of worthlessness, difficulty concentrating, psychomotor changes, and self-harm-related thoughts. Responses to each item were recorded from 0 to 3 according to symptom frequency, yielding a total score ranging from 0 to 27. Higher scores indicate greater depressive symptom severity. Based on established cutoff values, participants were classified into minimal, mild, moderate, moderately severe, and severe symptom categories. To further characterize symptom burden at the population level, two binary outcome measures were derived, representing PHQ-9 screen-positive symptoms (score ≥5) and clinically relevant moderate-or-higher depressive symptoms (score ≥10).

### 2.4 Assessment of anxiety symptoms

Anxiety symptoms were assessed using the GAD-7 scale, a standardized instrument commonly used to identify and quantify symptoms of generalized anxiety (15). The scale evaluates seven anxiety symptom domains experienced during the previous 2 weeks, including feeling nervous or anxious, inability to control worrying, excessive worry, difficulty relaxing, restlessness, irritability, and fear that something awful might happen. Responses to each item were recorded from 0 to 3 according to symptom frequency, yielding a total score ranging from 0 to 21, with higher scores reflecting greater anxiety symptom burden. Following established scoring recommendations, participants were categorized into minimal, mild, moderate, and severe anxiety symptom groups. To facilitate population-level assessment of anxiety burden, two binary outcome measures were additionally derived, representing GAD-7 screen-positive symptoms (score ≥5) and clinically relevant moderate-or-higher anxiety symptoms (score ≥10).

### 2.5 Data processing

Data from the three survey waves were merged into a harmonized long-format dataset, with each record representing a single questionnaire response. To ensure comparability across survey years, variable names, response categories, and coding schemes were systematically standardized during preprocessing. All variable labels and categorical response options were translated into English, and corresponding numeric codes were created to facilitate statistical analyses and direct import into SPSS. Data cleaning procedures included verification of variable formats, inspection of missing values, consistency checks across survey waves, and generation of derived variables for mental health outcomes. PHQ-9 and GAD-7 total scores, symptom severity categories, and screening indicators were calculated according to predefined scoring criteria. Personal identifiers were removed, whereas pseudonymized participant identifiers were retained for potential longitudinal linkage and quality-control purposes. The final analytic file consisted of demographic characteristics, family background factors, lifestyle and behavioral indicators, PHQ-9 and GAD-7 item-level and total scores, severity classifications, screening outcomes, and missingness summaries. This harmonized dataset provides a standardized framework for descriptive epidemiological analyses and serves as a foundation for future multivariable modeling and longitudinal research.

### 2.6 Statistical analysis

Continuous variables are reported as mean ± standard deviations (SD) and categorical variables as n (%). Across-administration differences in age, PHQ-9, and GAD-7 were evaluated using Kruskal-Wallis tests because score distributions were non-normal. Epsilon-squared quantified effect size. Categorical differences were evaluated with chi-square tests and Cramer’s V. Effect sizes were interpreted as evidence of practical magnitude rather than relying solely on *p* values.

Because some students contributed observations to more than one administration, generalized estimating equations with exchangeable working correlation and robust standard errors were used for inferential comparisons. Gaussian GEE models estimated adjusted mean differences in PHQ-9 and GAD-7 scores, and binomial GEE models estimated odds ratios for moderate-or-higher symptoms and Item 9 endorsement. Models included survey administration, age, and sex. Internal consistency was evaluated using Cronbach’s alpha overall and within each administration. Convergent association between PHQ-9 and GAD-7 total scores was assessed using Spearman correlation. A standardized principal-component analysis of the 16 items was used as a supplementary dimensional summary; the first component explained 55.8% of total variance.

For focused analysis of Item 9, a multivariable binomial GEE model included survey administration, age, sex, family economic status, number of close friends, current smoking, current alcohol use, the PHQ-8 score (PHQ-9 excluding Item 9), and GAD-7 score. All tests were two-sided with alpha = 0.05. Analyses were performed using Python statistical libraries and independently checked against the SPSS-ready data structure.

## 3 Results

### 3.1 Data processing and analytic sample

Figure 1 summarizes the systematic data-processing workflow. Three raw survey datasets, corresponding to the 2023, 2024, and 2025 survey waves, were initially collated for integration. These files underwent a rigorous harmonization procedure that included the systematic standardization of variable names, response categories, and coding schemes across all three survey years. The final analytic dataset consisted of 4,236 questionnaire records comprising 62 variables, which comprehensively covered survey wave information, demographic characteristics, family background, behavioral and lifestyle factors, and item-level as well as aggregate mental health scores. This harmonized SPSS-ready analytic file provides a robust and standardized foundation for subsequent descriptive epidemiology, subgroup comparisons, and advanced multivariable modeling.

**Figure 1:**
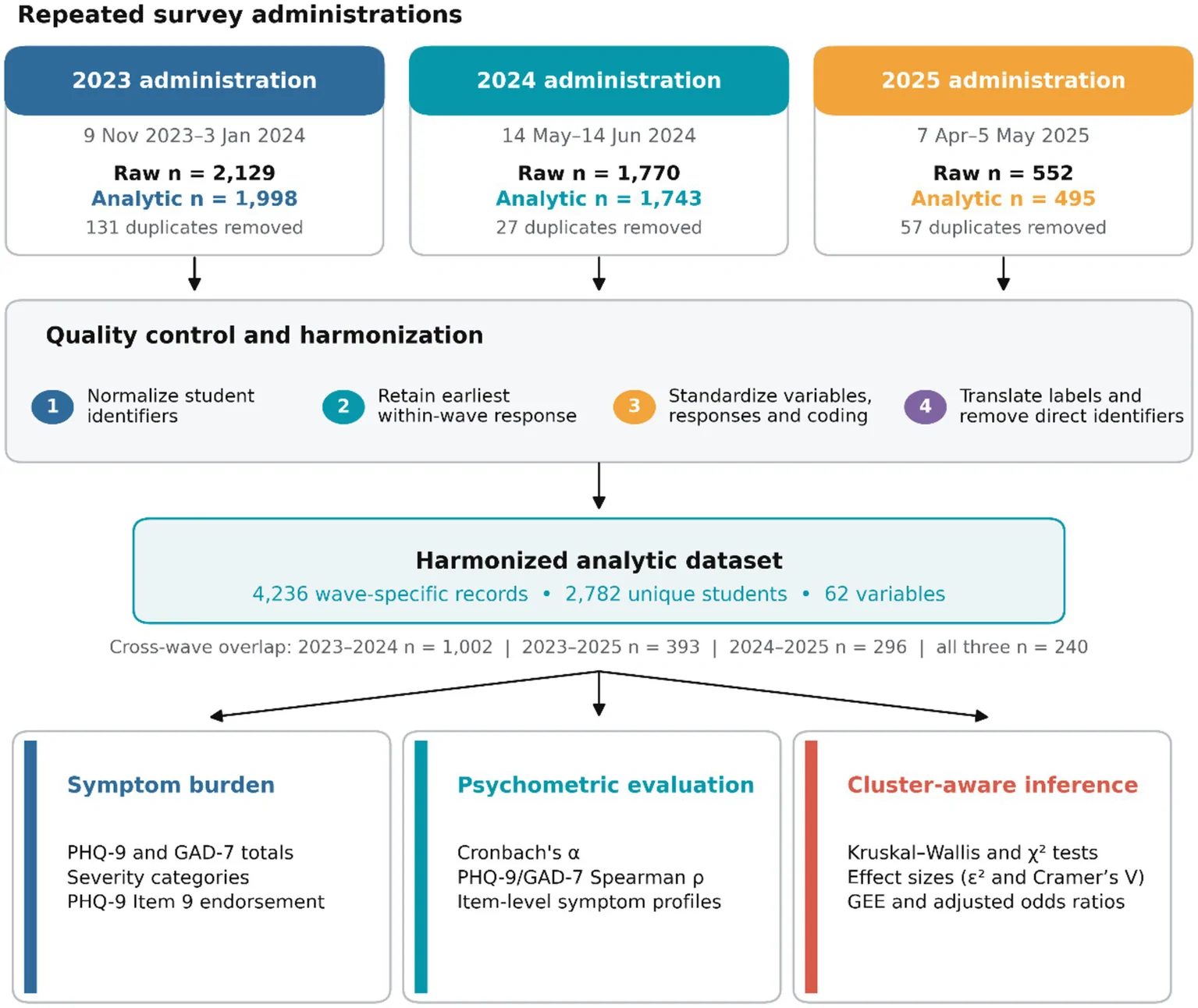
Study data processing flow.

### 3.2 Sample characteristics

A total of 4,236 participants were included across the three survey administrations. The overall mean age was 18.92 ± 0.77 years, and 59.4% of participants were female. Mean age increased progressively from 18.58 years in 2023 to 19.04 years in 2024 and 19.92 years in 2025 (*p* < 0.001), with a large effect size (epsilon-squared = 0.328), consistent with the expected aging of a predominantly 2023-entry cohort. Female participants accounted for 56.1, 62.7, and 61.6% of the samples in 2023, 2024, and 2025, respectively. Although sex distribution differed significantly across administrations (*p* < 0.001), the effect size was small (Cramer’s V = 0.066), indicating good comparability between survey waves.

Residential background also showed statistically significant variation (*p* = 0.009), with the proportion of rural students decreasing from approximately 44.5% in 2023–2024 to 36.6% in 2025, accompanied by a corresponding increase in urban residence. However, the associated effect size remained small (Cramer’s V = 0.040), suggesting that these differences were of limited practical importance. Economic status showed a modest statistical difference across administrations (*p* = 0.035; Cramer’s V = 0.040), whereas only-child status, number of close friends, current smoking, and current alcohol use did not differ significantly between survey waves (all *p* > 0.05). After correction of the alcohol-use coding error, the prevalence of current alcohol use was 11.8% in 2023, 12.9% in 2024, and 12.5% in 2025. Overall, despite several statistically significant differences attributable to the large sample size, the consistently small effect sizes indicate that participant characteristics were highly comparable across the three survey administrations (Table 1).

**Table 1:** Sample characteristics overall and by survey wave.

Characteristic	Overall	2023	2024	2025	*p* value	Effect size
Sample size, *n*	4,236	1998	1743	495	—	—
Age, years	18.92 ± 0.77	18.58 ± 0.65	19.04 ± 0.63	19.92 ± 0.70	<0.001	0.328
Female	2,518 (59.4%)	1,120 (56.1%)	1,093 (62.7%)	305 (61.6%)	<0.001	0.066
Rural residence	1848 (43.6%)	890 (44.5%)	777 (44.6%)	181 (36.6%)	0.009	0.040
Township residence	794 (18.7%)	384 (19.2%)	306 (17.6%)	104 (21.0%)	0.009	0.040
Urban residence	1,594 (37.6%)	724 (36.2%)	660 (37.9%)	210 (42.4%)	0.009	0.040
Only child	1,166 (27.5%)	565 (28.3%)	461 (26.4%)	140 (28.3%)	0.423	0.020
Poor/relatively poor economic status	1,196 (28.2%)	602 (30.1%)	463 (26.6%)	131 (26.5%)	0.035	0.040
Three to five close friends	1815 (42.8%)	873 (43.7%)	727 (41.7%)	215 (43.4%)	0.455	0.019
Current smoking	73 (1.7%)	32 (1.6%)	35 (2.0%)	6 (1.2%)	0.412	0.020
Current alcohol use	521 (12.3%)	235 (11.8%)	224 (12.9%)	62 (12.5%)	0.591	0.016

Values are mean ± SD or *n* (%). Continuous variables were compared using Kruskal-Wallis tests with epsilon-squared effect sizes; categorical variables were compared using chi-square tests with Cramer’s V.

### 3.3 PHQ-9 depressive symptoms

The overall mean PHQ-9 score was 5.08 ± 4.08, indicating a generally mild level of depressive symptoms in the study population (Figure 2; Table 2). Across the three survey administrations, the mean score increased from 4.85 ± 3.98 in 2023 to 5.46 ± 4.25 in 2024 before declining to 4.68 ± 3.81 in 2025. This pattern was consistent with the temporal trend illustrated in Figure 2, with the 2024 survey wave exhibiting the highest average depressive symptom burden. Although the difference among survey waves was statistically significant (*p* < 0.001), the corresponding effect size was very small (epsilon-squared = 0.006), indicating that the between-wave variation in mean symptom severity was modest.

**Figure 2:**
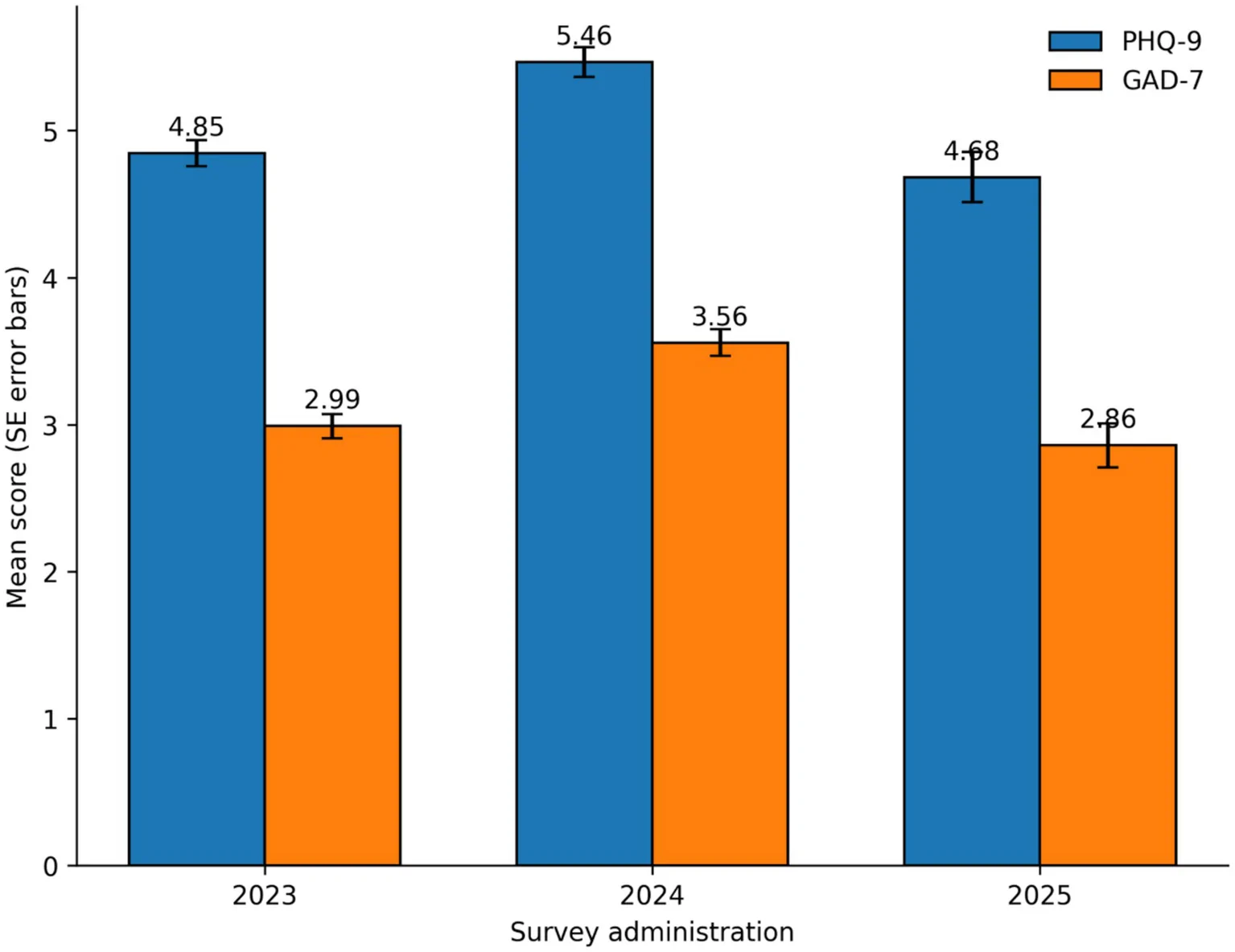
PHQ-9 and GAD-7 mean scores by survey administration; error bars are standard errors.

**Table 2:** Mental health outcomes overall and by survey wave.

Outcome	Overall	2023	2024	2025	*p* value	Effect size
PHQ-9 mean score	5.08 ± 4.08	4.85 ± 3.98	5.46 ± 4.25	4.68 ± 3.81	<0.001	0.006
PHQ-9 moderate or above (≥10)	365 (8.6%)	172 (8.6%)	166 (9.5%)	27 (5.5%)	0.017	0.044
Self-harm thoughts item >0	542 (12.8%)	195 (9.8%)	272 (15.6%)	75 (15.2%)	<0.001	0.086
GAD-7 mean score	3.21 ± 3.70	2.99 ± 3.67	3.56 ± 3.79	2.86 ± 3.36	<0.001	0.007
GAD-7 moderate or above (≥10)	209 (4.9%)	103 (5.2%)	94 (5.4%)	12 (2.4%)	0.022	0.042

*p* values compare the three waves. Effect size is epsilon-squared for continuous outcomes and Cramer’s V for categorical outcomes.

The distribution of PHQ-9 severity categories is shown in Figure 3. Across all survey administrations, minimal and mild depressive symptoms accounted for the majority of participants. In 2023, 48.9% of participants were classified as having minimal symptoms and 42.4% as having mild symptoms, whereas the corresponding proportions in 2024 were 43.4 and 47.1%, respectively. By 2025, the proportion of participants with minimal symptoms increased to 50.1%, while the proportion with mild symptoms decreased to 44.4%. Moderate depressive symptoms accounted for 6.3, 6.5, and 4.8% of participants in 2023, 2024, and 2025, respectively, whereas moderately severe and severe depression together represented only a small proportion of the study population across all survey waves (Figure 3). Using the conventional threshold for clinically relevant depressive symptoms (PHQ-9 ≥ 10), the overall prevalence of moderate-or-higher depressive symptoms was 8.6%. The prevalence remained similar between the 2023 (8.6%) and 2024 (9.5%) survey administrations before declining to 5.5% in 2025. Although these differences were statistically significant (*p* = 0.017), the effect size was small (Cramer’s V = 0.044), suggesting limited practical differences across survey waves.

**Figure 3:**
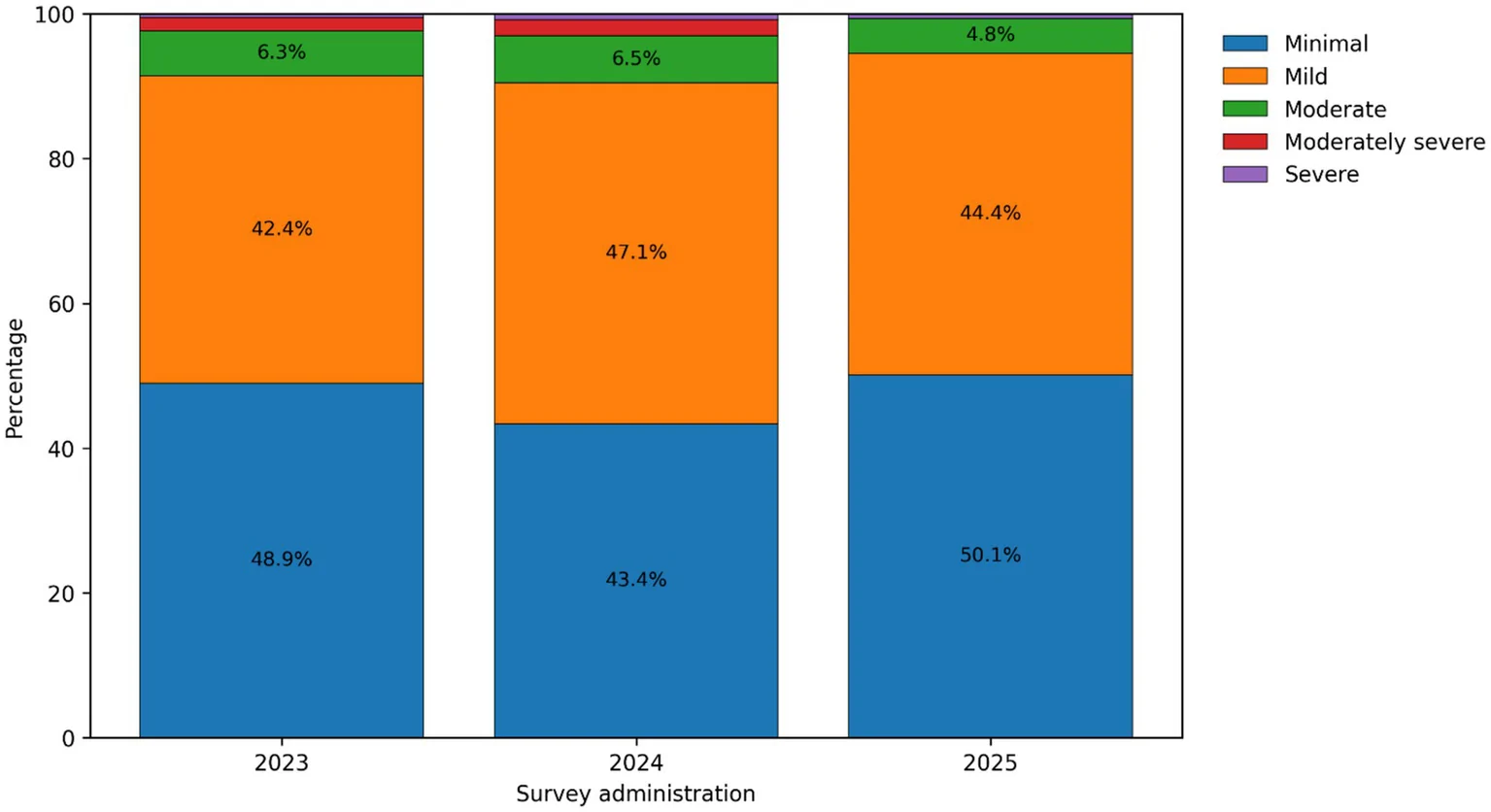
PHQ-9 severity distribution by survey administration.

The endorsement of the self-harm thoughts item (PHQ-9 Item 9 > 0) is summarized in Figure 4. Overall, 12.8% of participants endorsed Item 9. Wave-specific proportions increased from 9.8% in 2023 to 15.6% in 2024 and remained elevated at 15.2% in 2025. Among all depression-related outcomes, Item 9 demonstrated the largest between-wave difference (Cramer’s V = 0.086), although the effect size remained within the small range. Notably, despite the reduction in both the overall PHQ-9 mean score and the prevalence of moderate-or-higher depressive symptoms in 2025, the proportion of participants endorsing self-harm thoughts remained substantially higher than that observed in 2023, indicating that changes in suicidal ideation did not fully parallel changes in overall depressive symptom severity.

**Figure 4:**
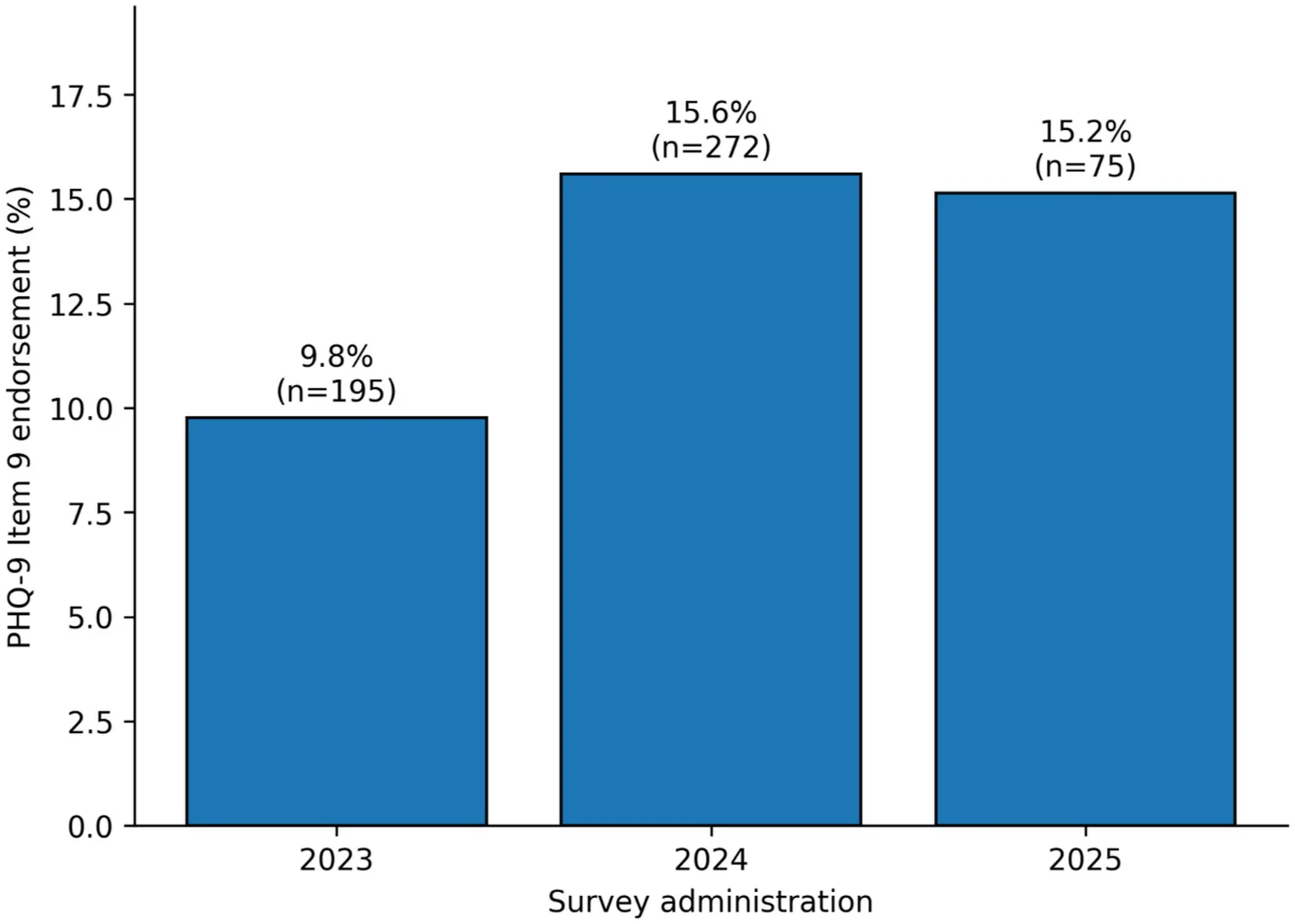
PHQ-9 Item 9 endorsement by survey administration.

### 3.4 GAD-7 anxiety symptoms

The overall mean GAD-7 score was 3.21 ± 3.70, reflecting a relatively low average anxiety symptom burden in the study population (Figure 2; Table 2). Consistent with the temporal pattern observed for depressive symptoms, the mean GAD-7 score increased from 2.99 ± 3.67 in 2023 to 3.56 ± 3.79 in 2024 before declining to 2.86 ± 3.36 in 2025. The 2024 survey administration exhibited the highest average anxiety symptom burden. Although the difference among survey waves was statistically significant (*p* < 0.001), the corresponding effect size was very small (epsilon-squared = 0.007), indicating only modest variation in average anxiety symptom severity across administrations.

The distribution of GAD-7 severity categories is presented in Figure 5. Across all three survey administrations, minimal anxiety symptoms represented the largest proportion of participants, accounting for 70.1% of the sample in 2023, 61.3% in 2024, and 68.1% in 2025. Correspondingly, mild anxiety symptoms were most prevalent during the 2024 survey wave (33.3%), compared with 24.8% in 2023 and 29.5% in 2025. Moderate anxiety symptoms accounted for only a small proportion of participants across all survey administrations, while severe anxiety remained uncommon throughout the study period (Figure 5). Overall, the distribution of anxiety severity shifted modestly toward higher symptom levels in 2024 and subsequently returned toward the 2023 pattern in 2025. Using the established threshold for clinically relevant anxiety symptoms (GAD-7 ≥ 10), the overall prevalence of moderate-or-higher anxiety symptoms was 4.9%. Wave-specific prevalence was 5.2% in 2023, 5.4% in 2024, and 2.4% in 2025. Although these differences reached statistical significance (*p* = 0.022), the corresponding effect size was small (Cramer’s V = 0.042), suggesting limited practical differences in the prevalence of clinically significant anxiety symptoms across survey administrations.

**Figure 5:**
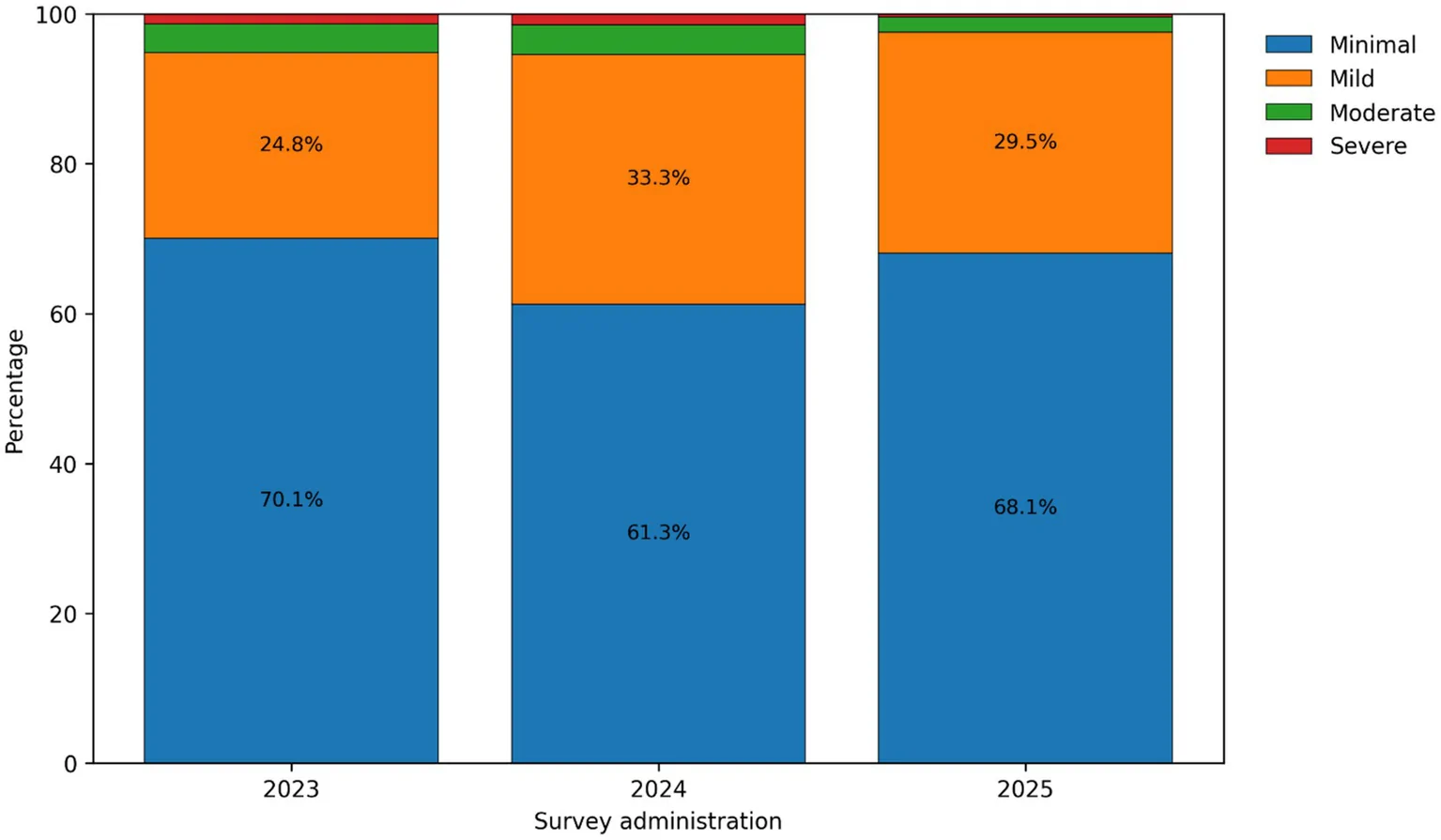
GAD-7 severity distribution by survey administration.

Overall, the temporal trends in GAD-7 scores closely paralleled those observed for PHQ-9, with both average symptom scores and severity distributions indicating a modest increase in symptom burden during the 2024 survey administration, followed by a decline in 2025. Nevertheless, the consistently small effect sizes for both continuous and categorical outcomes indicate that these between-wave differences were relatively modest and unlikely to represent substantial changes in the overall mental health profile of the surveyed student population.

### 3.5 Psychometric profiles

For the PHQ-9, the overall Cronbach’s α was 0.890, with administration-specific values of 0.873, 0.904, and 0.899 for the 2023, 2024, and 2025 survey waves, respectively. The GAD-7 showed similarly high reliability, with an overall Cronbach’s α of 0.937 and administration-specific values ranging from 0.929 to 0.943. These findings indicate that both scales maintained stable and robust internal consistency throughout the three survey administrations (Table 3). A strong positive association was observed between PHQ-9 and GAD-7 total scores. The overall Spearman correlation coefficient was ρ = 0.798 (*p* < 0.001), while administration-specific correlations ranged from 0.776 in 2023 to 0.815 in 2024, remaining consistently high across all survey waves (all *p* < 0.001). This pattern supports substantial convergent association between depressive and anxiety symptoms while indicating considerable overlap in the underlying psychological distress captured by the two instruments.

**Table 3:** Internal consistency and convergent association of the PHQ-9 and GAD-7.

Survey administration	*N*	PHQ-9 Cronbach α	GAD-7 Cronbach α	PHQ-9/GAD-7 Spearman ρ	*p* value
Overall	4,236	0.890	0.937	0.798	<0.001
2023	1998	0.873	0.929	0.776	<0.001
2024	1743	0.904	0.943	0.815	<0.001
2025	495	0.899	0.943	0.807	<0.001

Cronbach alpha was calculated using complete item responses. Spearman correlation assessed the association between total PHQ-9 and GAD-7 scores.

Item-level symptom profiles are summarized in Figure 6. Across all three survey administrations, fatigue (PHQ-4) consistently exhibited the highest mean score among PHQ-9 items (0.80, 0.82, and 0.75 in 2023, 2024, and 2025, respectively), followed by anhedonia (PHQ-1) and difficulty concentrating (PHQ-7), indicating that reduced energy, diminished interest, and concentration problems represented the most prominent depressive symptom domains. Sleep problems (PHQ-3) and appetite changes (PHQ-5) also showed relatively high mean scores, particularly during the 2024 survey administration. In contrast, psychomotor changes (PHQ-8) and self-harm thoughts (PHQ-9) consistently had the lowest mean scores across all survey waves, despite a modest increase in self-harm item scores in 2024 and 2025 relative to 2023.

**Figure 6:**
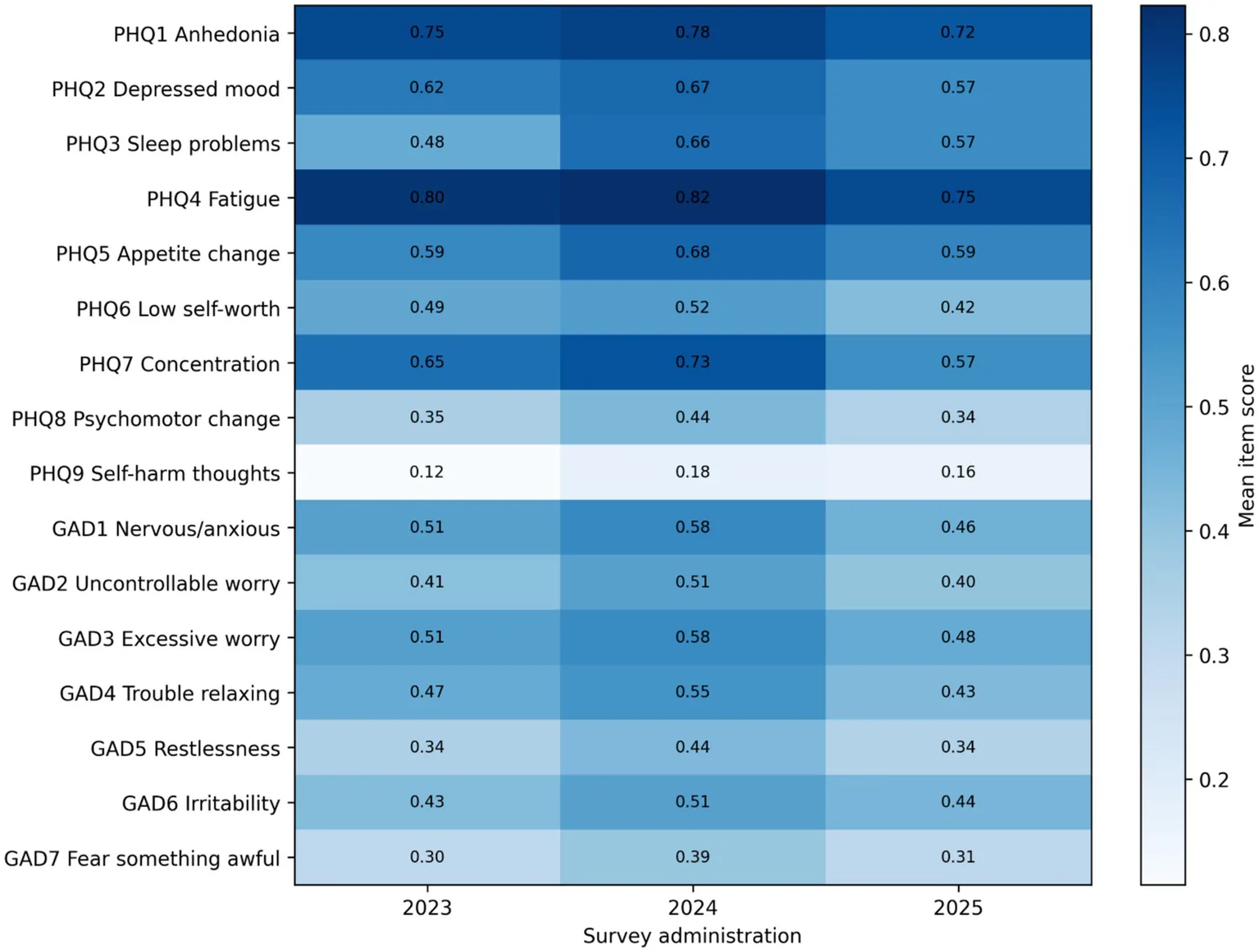
Item-level PHQ-9 and GAD-7 profiles.

Among the anxiety symptoms, feeling nervous or anxious (GAD-1) and excessive worry (GAD-3) consistently demonstrated the highest mean item scores, closely followed by difficulty relaxing (GAD-4) and uncontrollable worry (GAD-2). By comparison, restlessness (GAD-5) and fear that something awful might happen (GAD-7) showed the lowest average scores throughout the study period. Notably, nearly all PHQ-9 and GAD-7 items reached their highest mean values during the 2024 survey administration and subsequently declined in 2025, mirroring the temporal trends observed for the corresponding total scale scores. Collectively, these item-level findings suggest that the overall fluctuations in depression and anxiety scores across survey waves were broadly distributed across symptom domains rather than being driven by changes in a single specific symptom.

### 3.6 Item-level symptom patterns

PHQ-9 Item 9, indicating the presence of self-harm or suicidal thoughts, was endorsed by 542 of 4,236 observations (12.8%) overall. The prevalence increased from 9.8% in the 2023 survey administration to 15.6% in 2024 and remained elevated at 15.2% in 2025 (Figure 5). Although these differences were statistically significant (*p* < 0.001), the corresponding effect size was small (Cramer’s V = 0.086), indicating a modest but consistent increase in Item 9 endorsement across survey administrations. To identify factors independently associated with Item 9 endorsement, a generalized estimating equation (GEE) logistic regression model was fitted to account for repeated observations from the same participants across survey waves. After adjustment for demographic characteristics, lifestyle factors, depressive symptom severity excluding Item 9 (PHQ-8), and anxiety symptom severity (GAD-7), survey administration remained independently associated with self-harm thoughts. Compared with the 2023 survey administration, the adjusted odds of endorsing Item 9 were 50% higher in 2024 (adjusted OR = 1.50, 95% CI 1.17–1.92, *p* = 0.001) and 83% higher in 2025 (adjusted OR = 1.83, 95% CI 1.25–2.67, *p* = 0.002). Age showed a positive but non-significant association with Item 9 endorsement (adjusted OR = 1.17 per year, 95% CI 0.99–1.39, *p* = 0.072).

Among the remaining covariates, current alcohol use was independently associated with a greater likelihood of endorsing Item 9 (adjusted OR = 1.54, 95% CI 1.14–2.09, *p* = 0.005). Furthermore, both overall depressive symptom severity, represented by the PHQ-8 score (excluding Item 9), and anxiety symptom severity, represented by the GAD-7 total score, were significant independent correlates. Each one-point increase in PHQ-8 score was associated with a 15% increase in the odds of Item 9 endorsement (adjusted OR = 1.15, 95% CI 1.10–1.20, *p* < 0.001), whereas each one-point increase in GAD-7 score corresponded to a 29% increase in the odds (adjusted OR = 1.29, 95% CI 1.23–1.35, *p* < 0.001).

In contrast, sex, economic status, number of close friends, and current smoking were not independently associated with Item 9 endorsement after multivariable adjustment (all *p* > 0.05). Overall, the multivariable model suggests that the elevated prevalence of self-harm thoughts observed in the later survey administrations persisted after adjustment for demographic and behavioral characteristics and was primarily associated with greater depressive and anxiety symptom severity, together with current alcohol use (Figure 7; Table 4).

**Figure 7:**
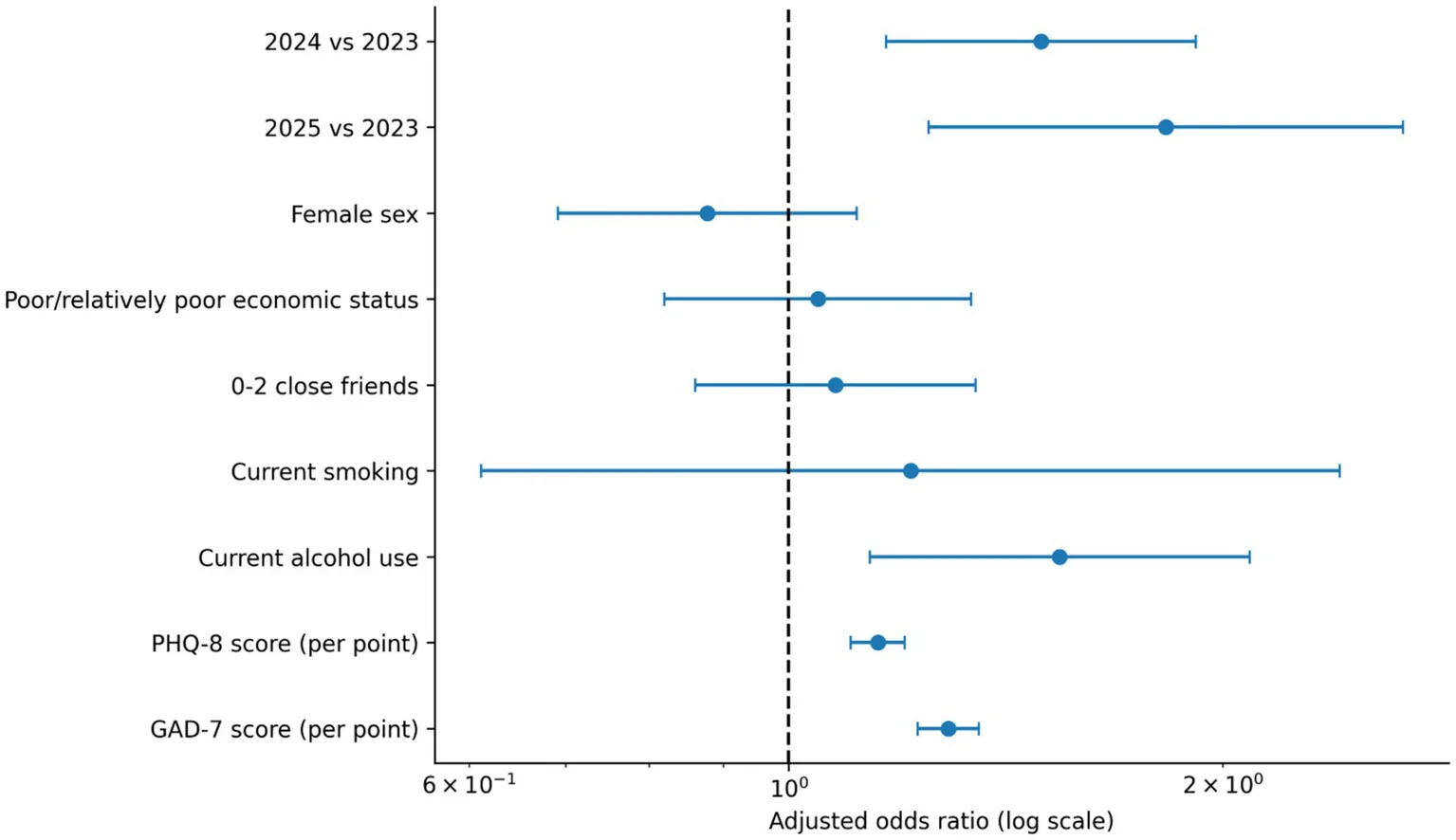
Adjusted correlates of PHQ-9 Item 9 endorsement.

**Table 4:** Multivariable correlates of PHQ-9 Item 9 endorsement.

Predictor	Adjusted OR (95% CI)	*p* value
2024 vs. 2023	1.50 (1.17–1.92)	0.001
2025 vs. 2023	1.83 (1.25–2.67)	0.002
Age (per year)	1.17 (0.99–1.39)	0.072
Female sex	0.88 (0.69–1.11)	0.285
Poor/relatively poor economic status	1.05 (0.82–1.34)	0.711
0–2 close friends	1.08 (0.86–1.35)	0.515
Current smoking	1.21 (0.61–2.41)	0.579
Current alcohol use	1.54 (1.14–2.09)	0.005
PHQ-8 score (per point)	1.15 (1.10–1.20)	<0.001
GAD-7 score (per point)	1.29 (1.23–1.35)	<0.001

Generalized estimating equation logistic model with exchangeable correlation and robust standard errors, accounting for repeated observations of the same student across survey administrations. PHQ-8 excludes Item 9.

## 4 Discussion

This three-wave serial cross-sectional investigation, encompassing 4,236 harmonized records, provides a robust descriptive characterization of the mental health burden among Chinese college students. Our findings yield several salient observations. First, subthreshold depressive and anxiety symptoms demonstrated a substantial prevalence, with 52.8 and 33.7% of the cohort exceeding the PHQ-9 and GAD-7 screening thresholds, respectively. Second, clinically significant symptom burdens (PHQ-9 ≥ 10: 8.6%; GAD-7 ≥ 10: 5.0%) and self-harm ideation (PHQ-9 Item 9: 12.8%) affected a smaller yet clinically critical subgroup. Third, a notable temporal fluctuation was observed, with the 2024 wave exhibiting the highest mean total scores and prevalence rates across both instruments, followed by a moderate decline in 2025. Finally, item-level analyses identified somatic symptoms (fatigue, sleep disturbances) and cognitive-affective deficits (anhedonia, concentration difficulties, excessive worry) as the predominant drivers of overall psychological distress.

The remarkably high prevalence of mild depressive and anxiety symptoms underscores a critical public health issue within the collegiate setting: emotional distress constitutes a normative transitional challenge for a significant proportion of students rather than an isolated phenomenon (16, 17). While these subthreshold symptoms may not fulfill the diagnostic criteria for major depressive disorder or generalized anxiety disorder, they represent a pivotal “window of opportunity” for early intervention. Indeed, subthreshold syndromes are well-established precursors to syndromal disorders, with well-documented detrimental impacts on academic engagement, social functioning, and quality of life (18, 19). Consequently, our findings substantiate the imperative for a tiered, stepped-care campus mental health framework. At the universal level, systematic interventions, such as sleep hygiene education, facilitated physical activity, and curriculum-integrated psychological literacy can benefit the broader student body (20, 21). For the screen-positive mild-symptom cohort, low-threshold supportive services, including peer support networks and digital self-help programs, may suffice to avert symptom progression (22, 23). However, the 8.6 and 5.0% of students exhibiting moderate-to-severe depressive and anxiety symptoms, respectively, alongside the 12.8% endorsing self-harm ideation, necessitate timely referral to specialized mental health professionals for evidence-based assessment and targeted psychotherapeutic or pharmacological management.

A striking finding was the uniform elevation of both depression and anxiety scores during the 2024 wave, a pattern consistently mirrored in the item-level profiles. It is crucial to recognize that the observed temporal peak was not confined to the 2024 survey; rather, it reflects a potential “period effect” superimposed on the cohort’s aging process. Attributing this fluctuation to a single causal factor would be premature given the descriptive, repeated cross-sectional design. Potential contributors may include acute academic stressors (e.g., intensive examination periods or graduation pressures), seasonal affective influences, or broader sociocultural shifts that were not explicitly captured in our questionnaire (24, 25). Additionally, variations in sampling strategies and response behaviors across administrations may have contributed to this variation. Nevertheless, monitoring such year-to-year fluctuations allows university administrators and health services to dynamically allocate resources and preemptively prepare for surges in service demand (26). Disentangling age-related, cohort-related, and period-related effects will be essential for future longitudinal modeling, which is currently hindered by the structural limitation of the present study (27, 28).

Of profound clinical significance is the dissociation between aggregate symptom severity and safety indicators. Notably, although the mean PHQ-9 scores and the prevalence of moderate-to-severe depressive symptoms declined in the 2025 wave, the endorsement rate for Item 9 (self-harm/suicidal ideation) remained elevated (15.2%) compared to 2023 (9.8%). This persistent elevation, confirmed by the multivariable GEE model (adjusted OR for 2025 vs. 2023 = 1.83, 95% CI 1.25–2.67), suggests that suicidal ideation operates through distinct and potentially more complex psychological pathways than general depressive severity. It underscores that an improvement in aggregate mood scores does not automatically translate to a diminution of suicide risk, which may be influenced by cumulative stress, interpersonal dynamics, or hopelessness that persist beyond overt mood symptoms (29). Therefore, these data powerfully advocate for the routine, independent screening for suicidal ideation in campus mental health surveillance, separate from general depression screening.

The item-level heatmap offers granular insights into specific symptom constellations that drive overall distress. The prominence of fatigue, anhedonia, and concentration difficulties within the PHQ-9, along with excessive worry and nervousness within the GAD-7, provides actionable targets for campus wellness programs (30). Interventions designed to improve circadian rhythms and sleep quality could directly address the high endorsement of fatigue and sleep disturbance (31, 32). Similarly, psychoeducational workshops focusing on cognitive-behavioral strategies for managing attention and worry, specifically tailored to the academic environment could prove particularly efficacious. Collectively, our findings highlight that a “one-size-fits-all” approach is insufficient; instead, targeted interventions that address the specific symptom profiles identified here are urgently required to optimize resource allocation and maximize clinical outcomes (33, 34).

### 4.1 Strengths and limitations

A strength of this study lies in its systematic and rigorous data harmonization across three distinct survey waves, which not only ensured uniformity in variable nomenclature, response coding, and missing value handling, but also established a reusable, SPSS-ready dataset that facilitates subsequent external validation and advanced analytical approaches such as machine learning modeling. Furthermore, the comprehensive reporting strategy, encompassing total scores, clinically relevant cutoffs, item-level profiles, and the independent assessment of self-harm ideation provides granular evidence to guide targeted campus interventions.

Several limitations should be noted. First, although the sample size was relatively large, the geographic confinement to a single university in China limits the generalizability of our findings to diverse academic environments, or to student populations in other regions with distinct sociocultural contexts and academic pressures (35, 36). Second, PHQ-9 and GAD-7 are validated screening tools, not diagnostic instruments; positive screens do not equate to clinical diagnoses, and the absence of structured interviews prevented assessment of functional impairment. Third, behavioral covariates (sleep, physical activity, sedentary time) were collected via coarse self-reports without objective validation, potentially introducing non-differential measurement error. Fourth, the fixed survey timing each academic year precluded adjustment for seasonal emotional fluctuations or examination-related stress cycles, which may have confounded wave-specific variations. Fifth, although student identifiers allowed quantification of participant overlap across administrations and enabled cluster-robust estimation, the design remains a repeated cross-sectional series with partial overlap rather than a fully longitudinal cohort with complete follow-up. Consequently, our data cannot empirically partition observed temporal fluctuations, such as the higher scores in 2024 or the persistent elevation of self-harm ideation in 2025 into age-related developmental changes, period-specific contextual factors, or cohort composition differences. With only three administrations and incomplete individual-level coverage, these competing explanations remain indistinguishable. Future prospective cohort studies with complete follow-up, ideally combined with age-period-cohort analytical frameworks, are needed to clarify the relative contributions of these temporal dimensions to college student mental health trajectories.

## 5 Conclusion

In these three repeated cross-sectional surveys of college students in Anhui Province, China, mild depressive and anxiety symptoms were highly prevalent, while moderate-to-severe symptoms impacted a smaller but clinically important subgroup. Critically, despite a decline in total mean symptom scores in 2025, the endorsement rates for self-harm ideation remained persistently elevated, indicating a dissociation between aggregate mood severity and suicide risk. Item-level analysis further identified fatigue, anhedonia, concentration difficulties, and excessive worry as the primary drivers of psychological distress. These findings substantiate the urgent need for a stratified campus mental health framework, encompassing universal health promotion, low-threshold supportive counseling, and timely specialized psychiatric referral, alongside routine, independent screening for suicidality. Moreover, the rigorously harmonized, SPSS-ready dataset serves as a reusable and robust foundation for future longitudinal modeling, risk-factor identification, and the translation of descriptive epidemiology into actionable, evidence-based mental health prevention.

## Data Availability

The raw data supporting the conclusions of this article will be made available by the authors, without undue reservation.
